# Polyvalent mRNA vaccine targeting outer surface protein C affords multi-strain protection against Lyme disease

**DOI:** 10.1038/s41541-025-01326-3

**Published:** 2025-12-04

**Authors:** Annabelle Pfeifle, Casey Lansdell, Wanyue Zhang, Levi A. Tamming, Rose Anderson-Duvall, Sathya N. Thulasi Raman, Caroline Gravel, Jianguo Wu, Grant Frahm, Marybeth Creskey, Maarten J. Voordouw, Heather Coatsworth, Weigang Qiu, Richard T. Marconi, Simon Sauve, Lisheng Wang, Xu Zhang, Michael J. W. Johnston, Xuguang Li

**Affiliations:** 1 Centre for Oncology, Radiopharmaceuticals and Research, Biologic and Radiopharmaceutical Drugs Directorate, Health Products and Food Branch, Health Canada and World Health Organization Collaborating Center for Standardization and Evaluation of Biologicals, Ottawa, ON Canada; 2 Department of Biochemistry, Microbiology and Immunology, Faculty of Medicine, University of Ottawa, Ottawa, ON Canada; 3 Department of Veterinary Microbiology, Western College of Veterinary Medicine, University of Saskatchewan, Saskatoon, SK Canada; 4 National Microbiology Laboratory, Public Health Agency of Canada, Winnipeg, MB Canada; 5 Department of Biological Sciences, Hunter College of City University of New York, New York, NY USA; 6 Department of Microbiology and Immunology, Virginia Commonwealth University, Richmond, Virginia USA; 7 Department of Chemistry, Carleton University, Ottawa, ON Canada

**Keywords:** Immunology, Microbiology

## Abstract

There is currently no Lyme disease (LD) vaccine available for use in humans. Outer surface protein C (OspC) of the causative agent, *Borrelia burgdorferi*, is a promising LD vaccine target. However, the extensive genetic variation of OspC poses a challenge in affording broad protection. Here, we developed a monovalent mRNA vaccine encoding OspC type A and a polyvalent vaccine encoding OspC types A, C, I, K, and N. The monovalent vaccine conferred complete protection against homologous challenge in mice, inducing functional OspC-specific antibodies and CD4⁺ T cell responses. The polyvalent formulation elicited antibodies to all encoded OspC types and protected against strains expressing OspC types A, I, and K, but not C or N. Increasing the dose enhanced protection against the OspC type C strain. This study is the first demonstration of an effective OspC-targeted mRNA vaccine and supports the development of OspC-based vaccines for broad LD prevention.

## Introduction

Lyme disease (LD) is a significant public health concern in temperate regions of North America and Europe. It remains the most common vector-borne disease across both continents, with an estimated 480,000 cases occurring in North America and over 120,000 reported cases in Europe annually^1–5^. These case numbers represent a substantial increase in incidence since monitoring began, driven largely by the expansion of associated tick habitats and greater human encroachment into endemic regions^6–8^.

LD is caused by spirochetes of the *Borrelia burgdorferi* sensu lato (sl) genospecies complex. The genospecies that cause most cases of LD are *B. burgdorferi* sensu stricto (ss) in North America and *B. afzelii* and *B. garinii* in Europe^9,10^. These spirochetes are maintained in nature via enzootic cycles involving wildlife hosts and ticks in the genus *Ixodes*, which transmit the spirochetes into the host skin. The bacteria then disseminate to various organs, including the joints, heart, and nervous system. In humans, *B. burgdorferi* ss infection initially presents with flu-like symptoms and the characteristic bull’s-eye rash, known as *erythema migrans*, in 70–80% of cases^11–13^. Severe manifestations of disseminated LD can include arthritis, carditis, and neurocognitive dysfunction^14–17^. Although antibiotics are available and effective for the treatment of LD, 10–20% of patients experience continued symptoms after appropriate treatment, termed post-treatment Lyme disease syndrome (PTLDS)^18–20^. Furthermore, many LD cases suffer from delayed diagnoses or misdiagnoses, emphasizing the need for improved preventative measures^21,22^.

LYMErix™, a recombinant subunit vaccine for LD, was previously approved by Health Canada and the United States Food and Drug Administration (FDA) in 1998^23^. The LYMErix™ vaccine targeted outer surface protein A (OspA), an immunogenic lipoprotein found on the surface of *B. burgdorferi*. Four years after approval, LYMErix™ was voluntarily withdrawn from the market by the manufacturer due to poor sales amidst reports of vaccine-induced arthritis, which were later unsubstantiated by an FDA review^23,24^. Since OspA is expressed by the spirochete during infection of the tick but is downregulated upon tick feeding and throughout mammalian infection, vaccines that target OspA must generate and maintain high levels of circulating antibodies that can enter the tick midgut during the tick blood meal to kill the spirochetes inside the tick before transmission to the host can occur^25–27^. As a result, annual booster injections were recommended for the LYMErix™ vaccine, introducing an additional burden on patients that may reduce vaccine compliance. A potentially more promising strategy for LD vaccine development is to target an alternative *B. burgdorferi* lipoprotein, such as outer surface protein C (OspC). In contrast to OspA, OspC is highly expressed during infection of the mammalian host and is required for early dissemination^25,28^. Vaccines targeting OspC may therefore require less frequent booster vaccinations^29^.

A major challenge in developing OspC-targeted vaccines is the high genetic diversity of the *ospC* gene within each *B. burgdorferi* sl genospecies. In North American strains of *B. burgdorferi* ss, the *ospC* gene is highly polymorphic with over 20 major allele groups that are referred to as OspC types or major groups^30–32^. Cross-protection between OspC types is limited, as previous infection or vaccination with recombinant OspC only affords protection against subsequent infection with homologous OspC types^33–36^. In areas where LD is endemic, human patients can be repeatedly infected with *B. burgdorferi* strains carrying different OspC types^37^. Additionally, studies examining *B. burgdorferi* strain diversity in populations of *Ixodes* ticks have repeatedly shown that a high percentage of infected ticks contain more than one OspC type^32,38–40^. An effective LD vaccine must therefore include a majority of the predominant OspC types in a given region.

The global deployment of the BNT162b2 and mRNA-1273 vaccines during the COVID-19 pandemic unequivocally demonstrated the immunogenicity, safety, and real-world efficacy of mRNA vaccines^41–43^. mRNA vaccines are highly adaptable and can be rapidly synthesized at large scales using cell-free systems. Recent research also suggests the utility of this platform to target bacterial infections, including two *B. burgdorferi* OspA-targeting vaccines undergoing preclinical evaluation^44–47^. Furthermore, the mRNA vaccine platform is particularly suited to the development of polyvalent vaccines, as mRNA encoding multiple antigens or antigen variants can be encapsulated within lipid nanoparticles (LNPs) and delivered together.

One clinically available veterinary vaccine consisting of a chimeric recombinant OspC (crOspC) protein in combination with OspA, Vanguard® crLyme, has demonstrated considerable efficacy and safety for the prevention of LD in canines^48^. Additionally, crOspC vaccines consisting of the immunodominant epitopes from a variety of different OspC types are capable of generating antibodies that recognize over 20 different OspC proteins spanning multiple *B. burgdorferi* genospecies and demonstrate borreliacidal activity in vitro^49–52^. Nevertheless, the breadth of protection conferred by OspC vaccines in the absence of OspA has not been thoroughly investigated.

Here, we developed both a monovalent and polyvalent nucleoside-modified mRNA-LNP vaccine encoding either OspC type A alone (OspC-A mRNA) or in conjunction with four other OspC types that are among the most prevalent in human and tick infections in North America (OspC-ACIKN mRNA)^32,53–55^. We first investigated the protection conferred by the monovalent vaccine against homologous *B. burgdorferi* needle challenge in a C3H/HeN mouse model. We then characterized the antibody and T cell immune responses induced by the monovalent OspC-A mRNA vaccine. Finally, we assessed the breadth of protection afforded by the polyvalent OspC-ACIKN mRNA against the five corresponding infection strains. Ultimately, our results demonstrate that OspC-targeting mRNA vaccines elicit both antigen-specific humoral and cellular-mediated immunity and can confer simultaneous protection against multiple *B. burgdorferi* strains.

## Results

### OspC-A mRNA affords complete protection against *B. burgdorferi* infection and dissemination

**Fig. 1:**
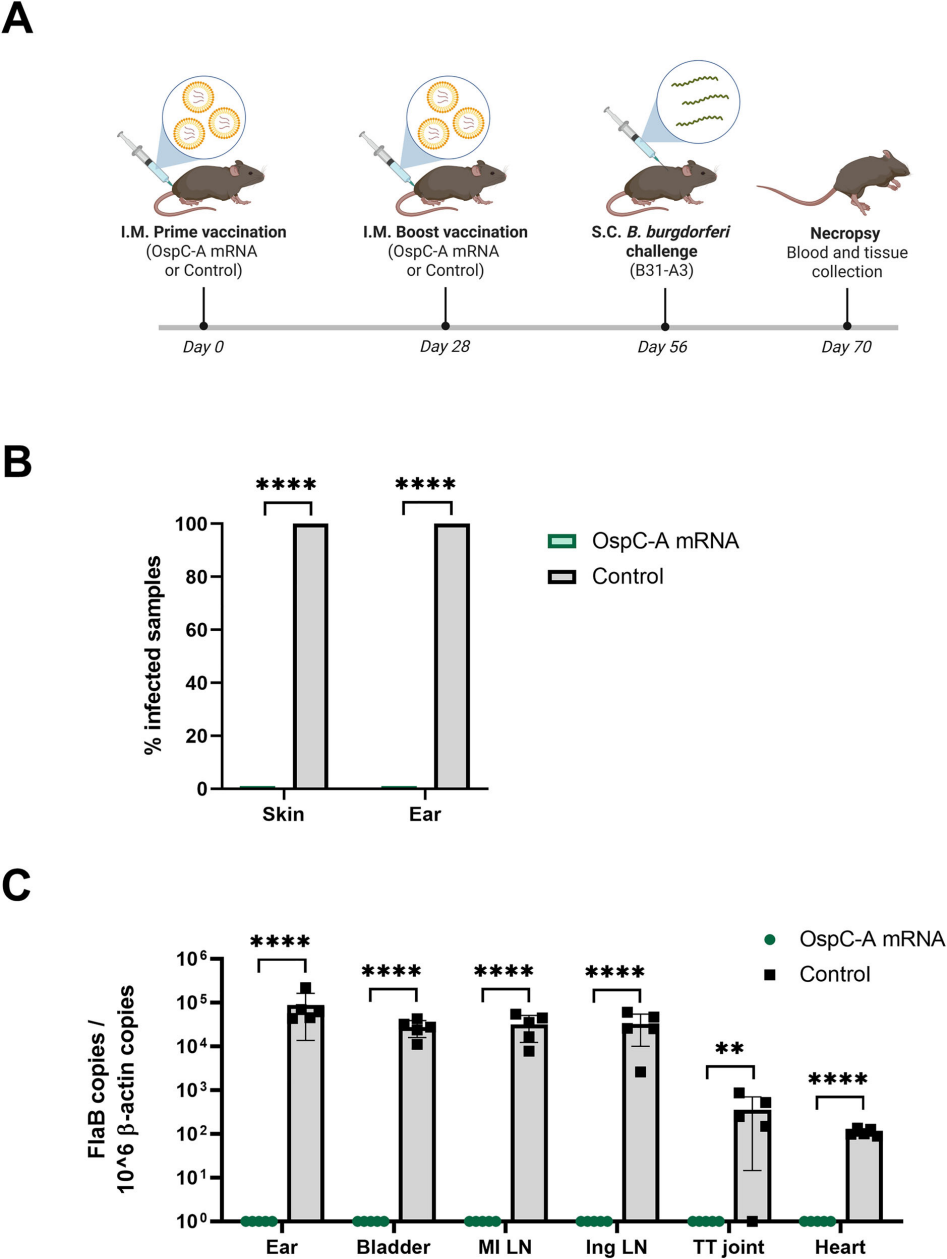
OspC-A mRNA affords protection against infection and dissemination with homologous *B. burgdorferi.* **A** Schematic depicting the procedure schedule for C3H/HeN mice. Mice were injected with the OspC-A mRNA vaccine or an empty LNP control (Control) on day 0 and 28 (*n* = 5 per group), challenged with homologous *B. burgdorferi* B31-A3 on day 56, and euthanized on day 70. Created using BioRender.com. **B** Percentage of infected mice following challenge. The skin surrounding the spirochete injection site and right ear were collected at necropsy and placed in BSK-H culture media for 28 days at 35 °C, 1.5% CO_2,_ before examination by dark-field microscopy. Samples were considered positive for infection if at least one spirochete was identified across five fields of view. Statistical significance was calculated using Fisher’s exact test. **C** qPCR analysis of the bacterial DNA burden represented by *B. burgdorferi* FlaB gene copies per 10^6^ mouse β-actin copies. Samples below the threshold of amplification are represented by a value of 1. TT joint, tibiotarsal joint; MI LN, medial iliac lymph node; Ing LN inguinal lymph node. Statistical significance was calculated using independent two-tailed *t*-tests. Error bars represent standard deviation. **p value < 0.01, ****p value <0.0001.

To evaluate the ability of the candidate mRNA vaccine to protect against *B. burgdorfer*i infection, female C3H/HeN mice were vaccinated with a prime-boost regimen and then challenged with a homologous strain of *B. burgdorferi*. On day 0, mice were injected intramuscularly with 1 µg of OspC-A mRNA or an empty LNP control containing an equivalent amount of lipid (Control). An identical boost injection was administered on day 28, and then 4 weeks later, mice were challenged subcutaneously with 10^6^ spirochetes expressing the matching OspC type A. 14 days later, mice were sacrificed for tissue collection (Fig. 1A).

Infection status of the mice was determined by culturing spirochetes from explants of the mouse skin and ears. The presence of motile spirochetes was detected in the samples using dark-field microscopy. All mice injected with the empty LNP control were positive for *B. burgdorferi* infection in both the skin and ears (Fig. 1B), demonstrating robust infection via the needle challenge. In comparison, all skin and ear samples collected from mice vaccinated with OspC-A mRNA were negative for the presence of spirochetes. Furthermore, qPCR was performed to quantify the bacterial burden across a wider array of tissues, including the ear, bladder, lymph nodes, tibiotarsal joint, and heart. Significant *B. burgdorferi* DNA loads were detected in all of these tissues in the control mice, whereas the bacterial DNA was below the limit of detection in all tissues of the OspC-A mRNA vaccinated mice (Fig. 1C). Taken together, these results suggest that the candidate OspC-A mRNA vaccine completely protects against infection and dissemination of *B. burgdorferi* strains carrying OspC type A in mice one month after boost vaccination.

### OspC-A mRNA protects against Lyme carditis and lymphadenopathy

**Fig. 2:**
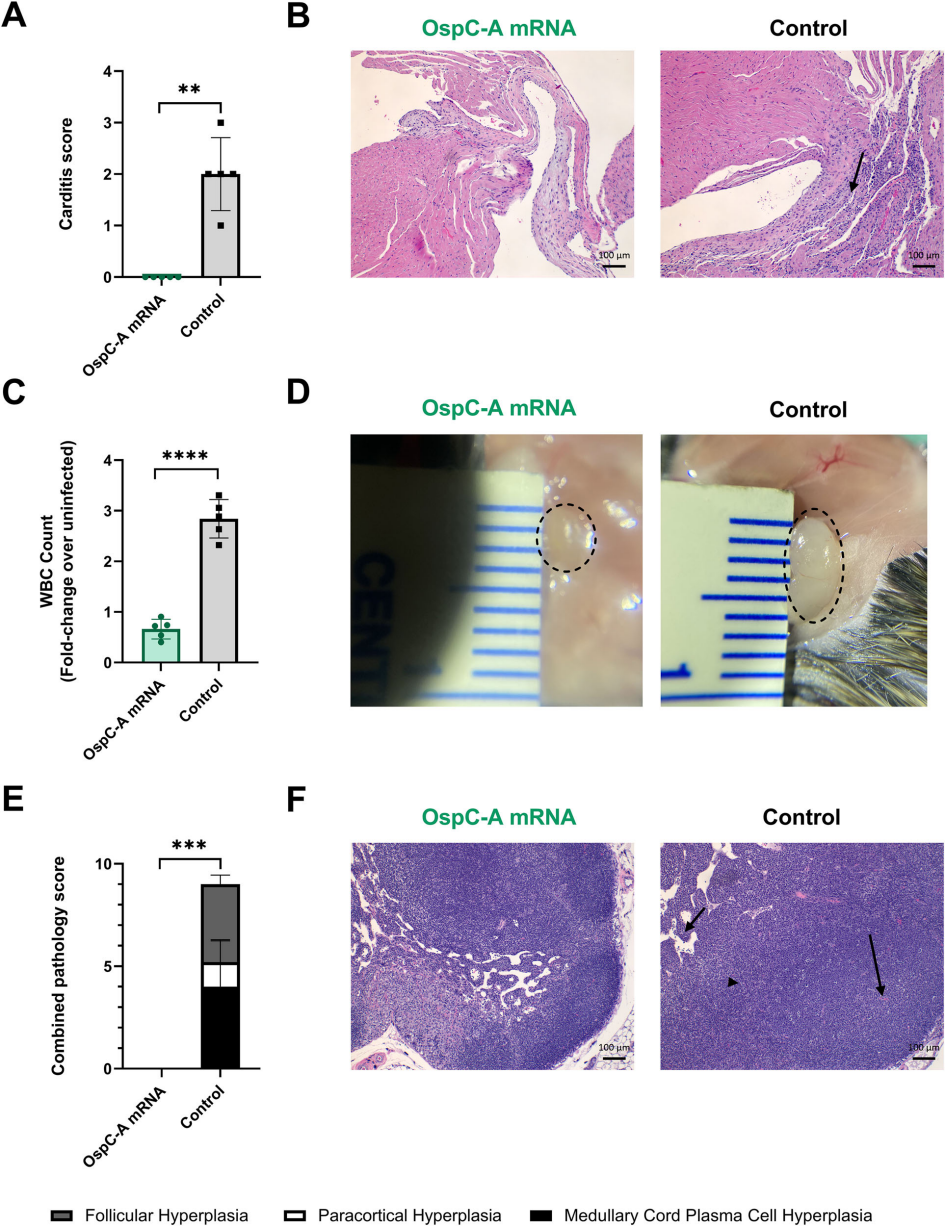
Vaccination with OspC-A mRNA protects mice against the development of *B. burgdorferi*-induced carditis and lymphadenopathy. **A** Summary of carditis pathology scores from mice vaccinated with OspC-A mRNA or the empty LNP control (Control). **B** Representative images of H&E-stained heart tissue from mice vaccinated with OspC-A mRNA or the empty LNP control (Control). The arrow indicates moderate mononuclear-neutrophilic inflammation in the heart base. **C** White blood cell (WBC) counts of the left axillary lymph node extracted from OspC-A mRNA vaccinated and empty LNP control mice 2 weeks after bacterial challenge, normalized to the WBC counts from unchallenged and uninfected mice. **D** In situ images of the left axillary lymph node from mice injected with OspC-A mRNA or the empty LNP control at the time of necropsy (day 70). **E** Summary of lymph node pathology scores. **F** Representative images of H&E-stained axillary lymph nodes from mice vaccinated with OspC-A mRNA or the empty LNP control. Arrows indicate marked follicular hyperplasia (long arrow), severe medullary cord hyperplasia (short arrow), and mild paracortical hyperplasia (arrowhead). *n* = 5 per group, error bars represent standard deviation. Statistical significance was calculated using independent two-tailed *t*-tests. **p value <0.01, ***p value <0.001, ****p value <0.0001.

Disseminated infection with *B. burgdorferi* causes multisystem inflammation in both humans and mice. In human cases, Lyme carditis can cause life-threatening dysfunction of the conduction system, such as atrioventricular blocks^56^. To evaluate whether the candidate vaccine could prevent the development of carditis in mice, histopathological analysis of the heart tissue was conducted at 14 days post-infection. Control mice developed mild carditis, characterized by mixed mononuclear-neutrophilic infiltration of the heart base with variable involvement of adjoining structures (Fig. 2A, B). In comparison, mice vaccinated with the OspC-A mRNA vaccine did not exhibit any detectable carditis.

Dissemination of *B. burgdorferi* to the lymph nodes causes extensive extrafollicular proliferation of B cells^57^. This accumulation of B cells leads to swelling of the lymph nodes systemically and especially close to the site of infection^58,59^. Accordingly, we observed severe enlargement of the axillary lymph nodes in all control mice, whereas all OspC-A mRNA vaccinated mice had lymph nodes of a similar size to uninfected mice. In addition, white blood cell (WBC) counts from axillary lymph nodes of control mice were approximately fourfold higher than the WBC counts from the OspC-A mRNA group (Fig. 2C, D). Inflammation of the axillary lymph nodes in the control mice was characterized by marked follicular and medullary cord plasma cell hyperplasia and mild paracortical hyperplasia (Fig. 2E, F). Conversely, hyperplasia was not detected in any OspC-A mRNA vaccinated mice. In summary, these data demonstrate that the candidate OspC-A mRNA vaccine completely protects mice against carditis and lymphadenopathy associated with *B. burgdorferi* infection.

### OspC-A mRNA generates a functional antibody response

**Fig. 3:**
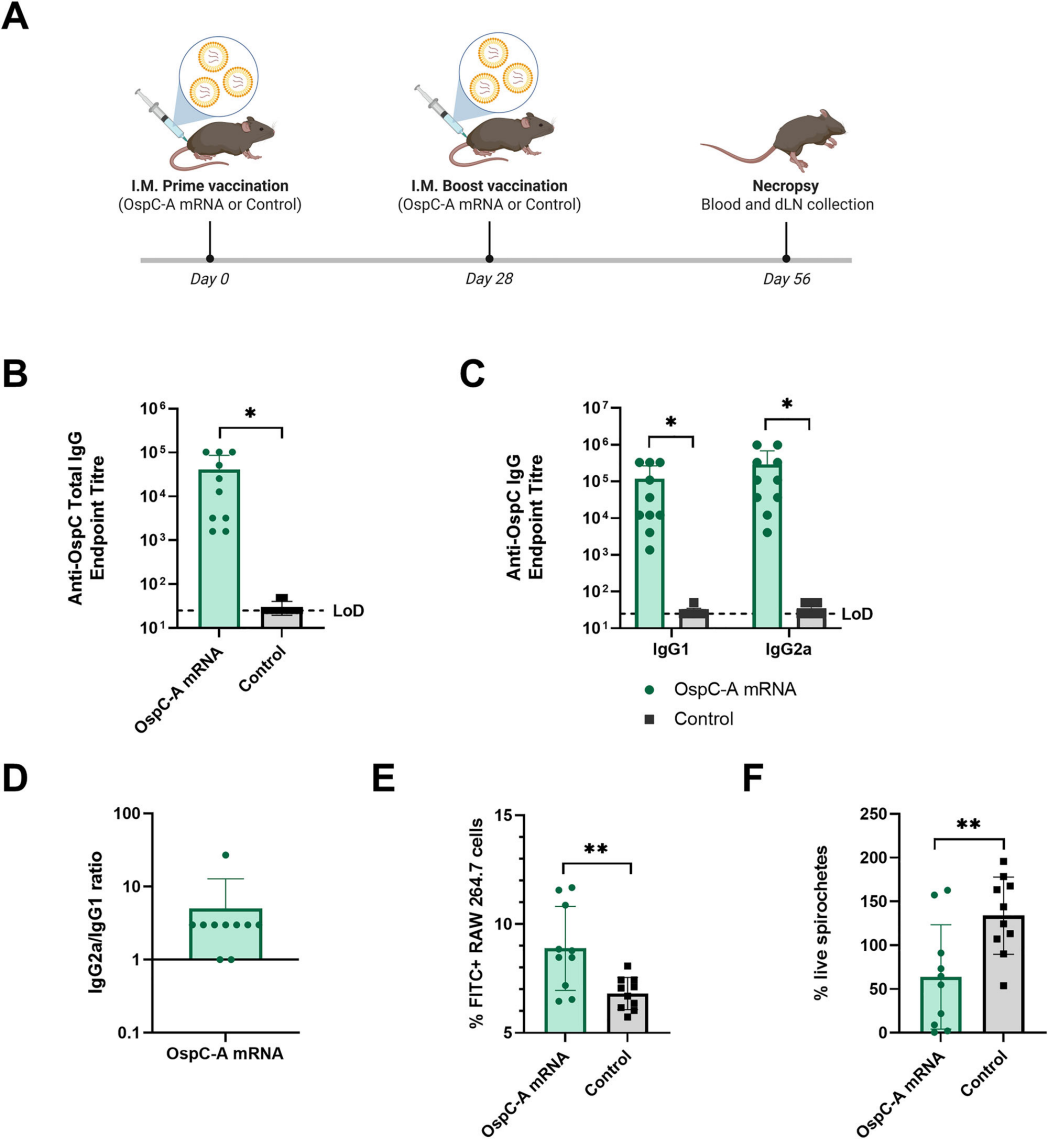
Vaccination with OspC-A mRNA induces a functional Th1-skewed anti-OspC antibody response in mice. **A** Schematic depicting the procedure schedule for C3H/HeN mice. Mice were injected with the OspC-A mRNA vaccine or luciferase mRNA control (Control) on day 0 and 28 (*n* = 10 per group) and euthanized on day 56 for collection of blood and draining lymph nodes (dLNs). Created using BioRender.com. ELISA quantification of **B** anti-OspC total IgG and **C** anti-OspC IgG1 and IgG2a antibody titers in the serum of mice at 4 weeks after boost vaccination. The dashed line indicates the limit of detection (LoD). Samples pertaining to data points on the dashed line have antibody titers below the limit of detection. **D** Ratio of IgG2a to IgG1 OspC-specific serum antibodies 4 weeks after boost vaccination. **E** Flow cytometry determination of the percentage of FITC + RAW 264.7 cells resulting from ADCP assay. **F** Percentage of live cells compared to no serum control following serum borreliacidal assay. Error bars represent standard deviation. Statistical significance was calculated using independent two-tailed *t*-tests. *p value <0.05, **p value <0.01.

To investigate the immunological mechanisms conferring the observed protection against *B. burgdorferi* infection and disease, mice were vaccinated with either the OspC-A mRNA vaccine or a negative control mRNA-LNP vaccine encoding firefly luciferase (Control). Mice were vaccinated with 1 µg of mRNA on days 0 and 28. Four weeks post-boost, the mice were euthanized without bacterial challenge (Fig. 3A).

Enzyme-linked immunosorbent assay (ELISA) was used to measure anti-OspC antibody titers in the serum of mice collected at 4 weeks post-boost. Vaccination with OspC-A mRNA produced significant anti-OspC antibody titers relative to the control (Fig. 3B). OspC-A mRNA vaccination induced both IgG1 and IgG2a subtypes, with an IgG2a:IgG1 ratio greater than 1 (average = 5 ± 7.8), indicating a potential Th1-biased immune response (Fig. 3C, D).

Complement-mediated killing and phagocytosis are two of the principal mechanisms of B. burgdorferi clearance by the immune system^60–63^. Antibody binding to B. burgdorferi surface proteins can mark the spirochetes for phagocytosis in a process referred to as opsonization or antibody-dependent cellular phagocytosis (ADCP). To evaluate whether the antibodies generated by vaccination with OspC-A mRNA were capable of inducing ADCP, fluorescently labeled *B. burgdorferi* were incubated with a RAW 267.4 mouse macrophage cell line in the presence of vaccinated serum. Since OspC is not highly expressed by B. burgdorferi in vitro, the B31-A3 strain used in this assay was engineered to constitutively express OspC (*B. burgdorferi-*OspC)^64,65^. Serum from mice vaccinated with OspC-A mRNA induced significantly higher levels of phagocytosis than serum from the control group, demonstrated by a greater percentage of fluorescent macrophages and a higher median fluorescence intensity (Fig. 3E). Additionally, incubation of B. burgdorferi-OspC with serum from mice vaccinated with OspC-A mRNA in the presence of guinea pig serum, resulted in a twofold decrease in live spirochetes after 24 h, compared to the control vaccine (Fig. 3F). Overall, these data suggest that the antibody response elicited by the candidate vaccine is functional and capable of inducing pertinent effector functions.

### OspC-A mRNA elicits antigen-specific CD4 + T cell responses

**Fig. 4:**
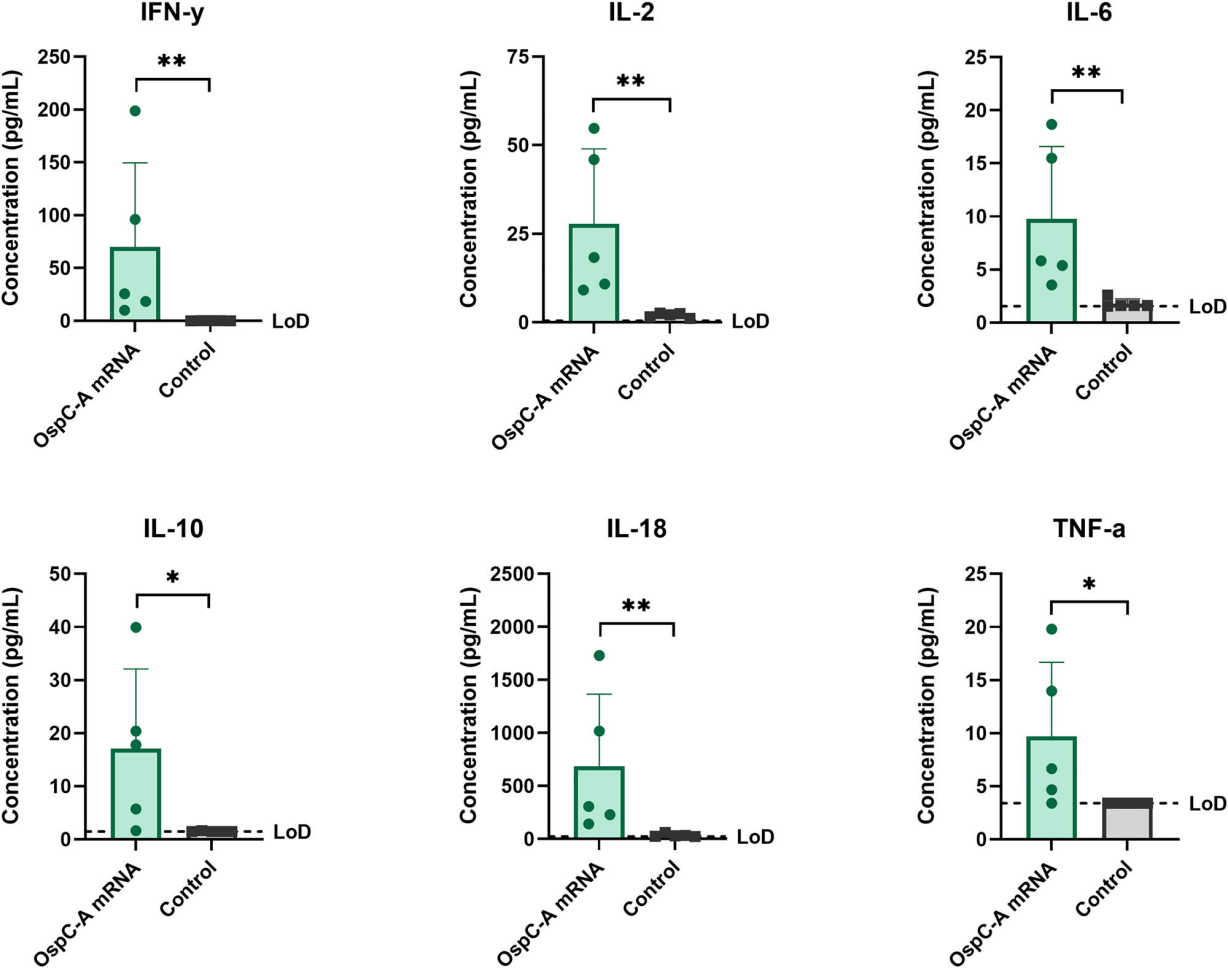
Vaccination of mice with OspC-A mRNA induces antigen-specific immune responses in the draining lymph nodes. dLNs were harvested from mice 4 weeks after boost vaccination and were stimulated with an overlapping peptide library of OspC type A for 24 h. The concentration of each cytokine in the supernatant was determined by the ProcartaPlex 17-plex Immunoassay kit. LoD, limit of detection. *n* = 5 per group, error bars represent standard deviation. Statistical significance was calculated using independent two-tailed *t*-tests. *p value <0.05, **p value <0.01.

**Fig. 5:**
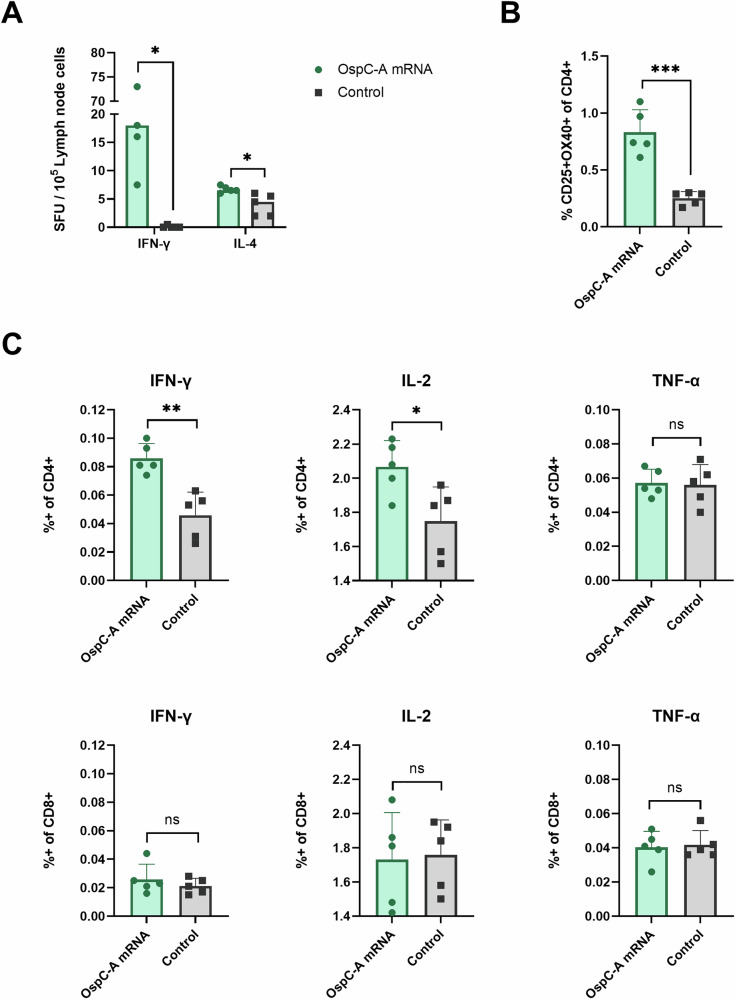
OspC-A induces OspC-specific T cell responses in the draining lymph nodes of OspC-A-vaccinated mice. **A** ELISpot determination of IFN-γ and IL-4 production by draining lymph node cells following 24 h stimulation with an overlapping OspC peptide pool represented by spot-forming units (SFU) per 10^5^ lymph node cells. **B** Flow cytometry analysis of activation-induced markers (AIM) expression (CD25 + OX40+) as a percentage of CD4 + T cells in the dLNs collected 4 weeks after boost vaccination following 18 h stimulation with an overlapping OspC peptide pool. **C** Flow cytometry analysis of the percentage of CD4+ (top) and CD8+ (bottom) T cells producing IFN-γ, IL-2, or TNF-α. *n* = 5 per group, error bars represent standard deviation. Statistical significance was calculated using independent two-tailed *t*-tests. ns not significant, *p value <0.05, **p value <0.01, ***p value <0.001.

Next, we assessed the cell-mediated immune response elicited by the OspC-A mRNA vaccine. Lymphocytes collected from the draining lymph nodes 4 weeks after the boost injection were stimulated with an overlapping peptide pool of OspC type A. Following stimulation, lymph node cells from OspC-A mRNA vaccinated mice secreted significantly higher levels of IFN-γ, IL-2, IL-6, IL-10, IL-18, and TNF-α than the control mice (Fig. 4). Secretion of Th2 cytokines, including IL-4, IL-5, and IL-13, was not elevated in the OspC-A mRNA group, nor was secretion of GM-CSF, IL-1β, IL-9, IL-12p70, IL-17A, IL-22, IL-23, and IL-24 (Fig. S1). ELISpot analysis of the stimulated lymph node cells detected considerable IFN-γ producing cells and minimal IL-4 producing cells in the OspC-A mRNA group compared to the control (Fig. 5A). In response to stimulation, OspC-A mRNA vaccinated mice had significantly higher populations of activated CD4 T cells (CD25 + OX40+) than control mice (Fig. 5B). Furthermore, intracellular cytokine staining demonstrated that CD4 + T cells but not CD8 + T cells produced IFN-γ and IL-2 following OspC-A mRNA vaccination (Fig. 5C). In conclusion, these findings provide robust evidence that the candidate OspC-A mRNA vaccine induces a strong antigen-specific Th1-biased CD4 + T cell response.

### Polyvalent OspC mRNA affords protection against multiple *B. burgdorferi* strains

**Fig. 6:**
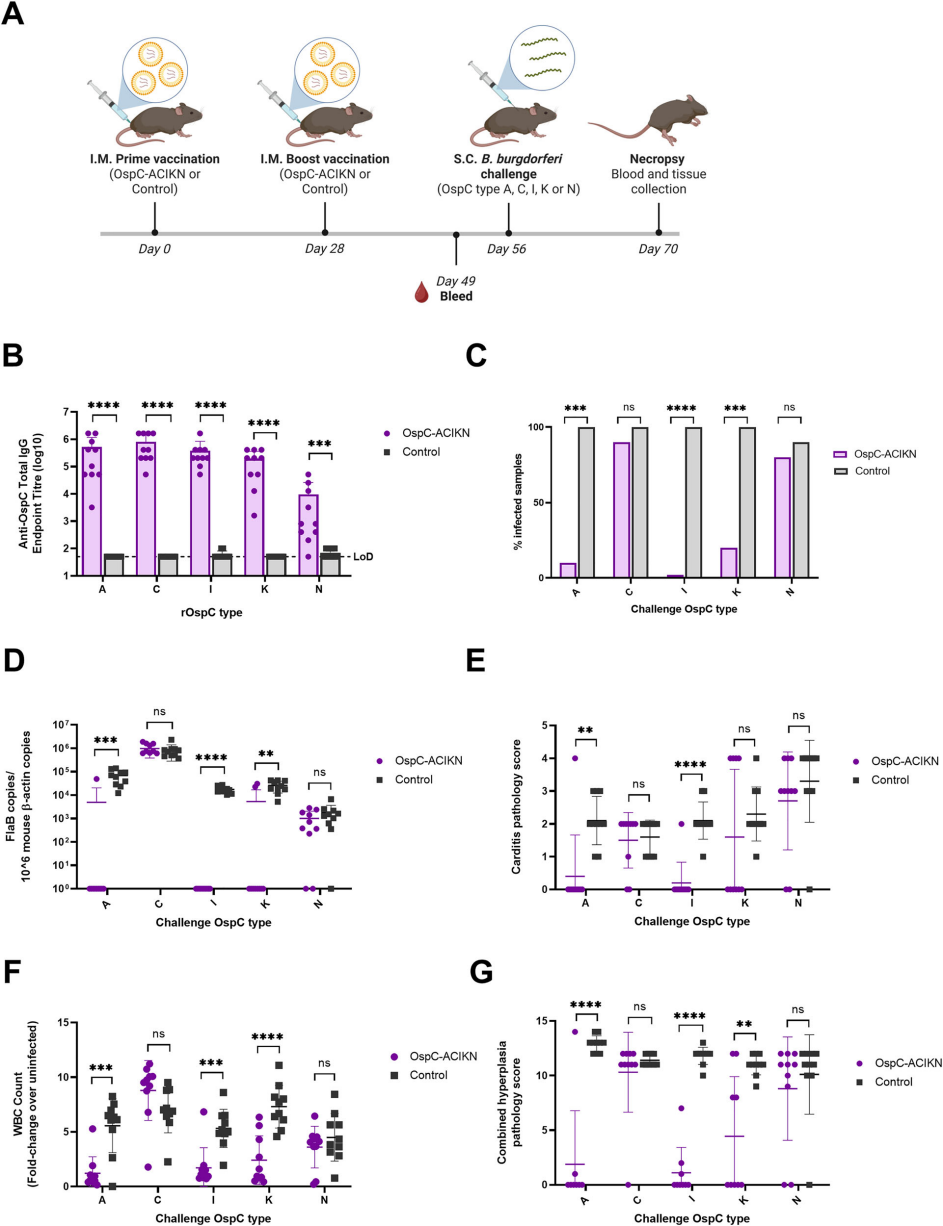
Vaccination with OspC-ACIKN mRNA protects mice against infection with multiple *B. burgdorferi* strains. **A** Schematic depicting the procedure schedule for C3H/HeN mice. Mice were injected with the OspC-ACIKN or luciferase mRNA vaccine on day 0 and 28, were bled on day 49, and challenged with either *B. burgdorferi* strain B31-A3 (OspC type A), JD1 (OspC type C), Bb16-54 (OspC type I), 297 Ah130 (OspC type K), or Bb16-126 (OspC type N) on day 56 (*n* = 10 per strain per vaccine group), and euthanized on day 70. Created using BioRender.com. **B** ELISA quantification of anti-OspC IgG antibody titers by recombinant OspC type in the serum of mice 3 weeks after boost vaccination and prior to challenge with the matching strain. **C** Percentage of infected mice following challenge. The skin surrounding the injection site was collected at necropsy and placed in BSK-H culture media for 28 days at 35 °C, 1.5% CO_2,_ before examination by dark-field microscopy. Samples were considered positive for infection if at least one spirochete was identified across five fields of view. **D** qPCR analysis of the bacterial DNA burden in the left ear represented by *B. burgdorferi* FlaB gene copies per 10^6^ mouse β-actin copies. Samples below the threshold of amplification are represented by a value of 1. **E** Summary of carditis pathology scores 2 weeks after challenge. **F** White blood cell (WBC) counts of the left axillary lymph node extracted from vaccinated and control mice 2 weeks after bacterial challenge, normalized to the WBC counts from unchallenged and uninfected mice. **G** Summary of lymph node pathology scores 2 weeks after challenge. Statistical significance was calculated using independent two-tailed *t*-tests for (**B**, **D**–**G**) and Fisher’s exact test for (**C**). Error bars represent standard deviation. ns not significant, **p value <0.01, ***p value <0.001, ****p value <0.0001.

To address the challenge presented by the genetic diversity of OspC, we generated a polyvalent mRNA vaccine encoding five of the most prevalent OspC types in North America, namely type A, C, I, K, and N (OspC-ACIKN)^32,36,54^. The mice were vaccinated in a prime-boost regimen with 1 µg of each OspC type or a negative control mRNA-LNP vaccine encoding firefly luciferase (Control), and then were challenged with one of the five *B. burgdorferi* strains carrying OspC types A, C, I, K, or N. Two weeks later, the mice were euthanized for tissue collection (Fig. 6A).

OspC-ACIKN vaccination induced significant serum IgG titers at 3 weeks after the boost against all included OspC types relative to the control (Fig. 6B). Following bacterial challenge, infection status was determined by culture of the skin surrounding the bacterial injection site for 28 days, and live spirochetes were identified by dark-field microscopy. 90–100% of control mice were positive for motile spirochetes in the skin across all *B. burgdorferi* strains (Fig. 6C). In comparison, only 10% of the mice vaccinated with OspC-ACIKN mRNA were positive for motile spirochetes with the type A strain, 20% were positive for the type K strain, and no mice vaccinated with OspC-ACIKN were positive for the type I strain. Interestingly, OspC-ACIKN vaccination only reduced infection with type C and type N strains by 10% compared to the control. These results were further confirmed by quantification of the *B. burgdorferi* DNA burden in the ear, bladder, tibiotarsal joint, and heart, which found that vaccination with OspC-ACIKN significantly reduced the tissue spirochete loads of the type A, I, and K strains but not the type C and N strains (Fig. 6D and Fig. S2).

We previously demonstrated that infection with each of the five *B. burgdorferi* strains causes inflammation of the heart base and hyperplasia of the axillary lymph nodes in mice at 2 weeks post-infection^66^. Here, we observed that the infection status, as determined by qPCR and explant culture, was associated with the development of carditis and lymphadenopathy. All control mice developed mild-to-marked Lyme carditis following infection with each strain (Fig. 6E). Additionally, OspC-ACIKN vaccinated mice with breakthrough infection of any strain developed minimal-to-marked carditis, with the exception of one mouse in the group challenged with the type C strain, which was previously shown to cause the lowest levels of carditis^66^. Overall, vaccination with OspC-ACIKN significantly reduced the development of Lyme carditis from infection with the type A and type I strains, but not types C, K, or N.

Similarly, control mice had severe lymph node enlargement and consistently developed moderate-to-severe lymph node hyperplasia following infection with all strains (Fig. 6F, G). Additionally, OspC-ACIKN vaccinated mice with breakthrough infection developed mild-to-severe lymph node hyperplasia. In comparison, OspC-ACIKN vaccinated mice that were protected from infection had significantly reduced lymph node cellularity and hyperplasia. Vaccination with OspC-ACIKN therefore reduced lymph node pathology resulting from infection with *B. burgdorferi* strains type A, I, and K, but not C and N. Overall, the OspC-ACIKN vaccine afforded significant protection against pathologies associated with infection of three of the five *B. burgdorferi* strains.

### Increasing vaccine dose improves protection against *B. burgdorferi* expressing OspC type C

Given that OspC-ACIKN vaccination of the mice provided minimal protection against *B. burgdorferi* strains with OspC types C and N, we performed a follow-up study to evaluate whether increasing the vaccine dose from 1 to 3 µg of mRNA would better prevent infection. In this study, mice were vaccinated with 3 µg of mRNA of each of the five OspC types or the luciferase mRNA as a control. Mice were bled for serum collection and then challenged 4 weeks post-boost with *B. burgdorferi* strain OspC type C, as this strain resulted in the highest rates of infection in mice vaccinated with 1 µg of OspC-ACIKN (Fig. 6C). Finally, the mice were euthanized on day 70 for tissue collection.

**Fig. 7:**
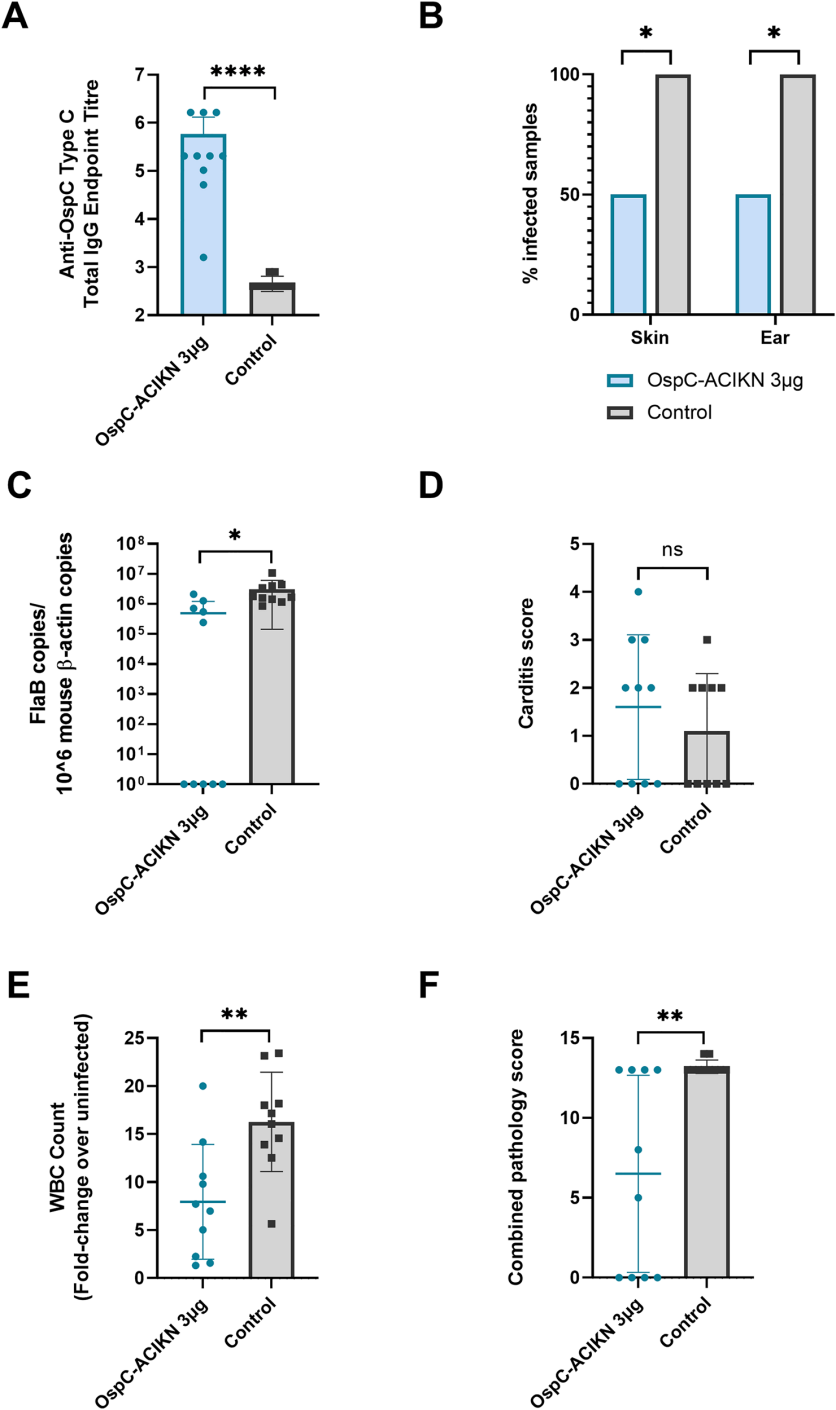
Vaccination of mice with a higher dose of OspC-ACIKN mRNA affords greater protection against infection with *B. burgdorferi* expressing OspC type C. **A** ELISA quantification of anti-OspC type C IgG antibody titers in the serum of mice 3 weeks after boost vaccination with a 3 µg dose of OspC-ACIKN or luciferase mRNA. **B** Percentage of infected mice following challenge. Two weeks after challenge with the *B. burgdorferi* OspC type C strain, the right ear and skin surrounding the injection site were collected and placed in BSK-H culture media for 28 days at 35 °C, 1.5% CO_2,_ before examination by dark-field microscopy. Samples were considered positive for infection if at least one spirochete was identified across five fields of view. **C** qPCR of the bacterial DNA burden in the left ear represented by *B. burgdorferi* FlaB gene copies per 10^6^ mouse β-actin copies. Samples below the threshold of amplification are represented by a value of 1. **D** Summary of carditis pathology scores 2 weeks after challenge with *B. burgdorferi* OspC type C strain. **E** White blood cell (WBC) counts of the left axillary lymph node extracted from vaccinated mice 2 weeks after type C challenge, normalized to the WBC counts from uninfected mice. **F** Summary of lymph node pathology scores 2 weeks after type C challenge. *n* = 10 per group, error bars represent standard deviation. Statistical significance was calculated using independent two-tailed *t*-tests for (**A**, **C**–**F**) and Fisher’s exact test for (**B**). ns not significant, * *p* value <0.05, **p value <0.01, ***p value <0.001, ****p value <0.0001.

The 3 µg dose of OspC-ACIKN mRNA induced significant IgG antibody titers in the serum 3 weeks post-boost when compared to the control (Fig. 7A). Moreover, bacterial culture from the right ear and skin surrounding the spirochete injection site demonstrated that vaccination with OspC-ACIKN protected 50% of the mice from infection with the type C strain (Fig. 7B), an increase relative to the 10% protection observed at the 1 µg dose (Fig. 6C). This finding was further confirmed by qPCR, which demonstrated a significant reduction in spirochete DNA of the type C strain in OspC-ACIKN vaccinated mice compared to control mice (Fig. 7C).

Infection with the OspC type C strain did not cause detectable carditis in half of the control mice (Fig. 7D). We have previously reported that the OspC type C strain causes the least carditis of the five strains evaluated in this study^66^. Consequently, vaccination with OspC-ACIKN did not significantly reduce inflammation in the heart compared to the control mice. In contrast, following the type C challenge, control mice developed significant hyperplasia of the axillary lymph nodes, as demonstrated by the substantially elevated white blood cell counts (Fig. 7E) and histopathological analysis (Fig. 7F). Mice vaccinated with 3 µg of OspC-ACIKN had significantly lower white blood cell counts and less hyperplasia of the axillary lymph nodes compared to the control group. Taken together, these results demonstrate that increasing the vaccination dose of mRNA can lead to improved protection against infection and pathology with the type C strain.

## Discussion

The causative agent of LD, *B. burgdorferi*, is estimated to infect nearly half a million individuals in North America each year^1,2,5^. Climate change models project that by 2050, Canada will have 120,000 to 500,000 cases of LD per year^67^. Despite the availability and efficacy of oral and intravenous antibiotic treatments, 10–20% of these patients will develop PTLDS or Lyme infection-associated chronic illnesses (IACI) significantly reducing their quality of life^18,68–70^. In the United States, LD, PTLDS, and Lyme IACI cases are a substantial economic burden, costing the healthcare system between 700 million and 1.3 billion USD annually^71^. For Canada, climate change models suggest that the economic costs of LD in 2050 could range between 0.5 and 2.0 billion Canadian dollars per year^67^. Vaccines are therefore an essential component of a comprehensive prevention plan for Lyme disease.

Since the removal of LYMErix™ in 2002, there is no option to vaccinate against LD in humans. LYMErix™, as well as the current clinical trial candidate, VLA15, are both recombinant protein vaccines targeting OspA^23,72^. Most preclinical and veterinary vaccine development efforts have focused on recombinant protein or subunit vaccine platforms. Logistically, mRNA vaccine production is more rapid and scalable than protein purification and mRNA vaccines facilitated the rapid response to the COVID-19 pandemic^73^. The ionizable lipid component of LNPs has been shown to have an adjuvanting effect on T follicular helper cells, thereby increasing antibody production^74^. Moreover, nucleoside-modified mRNA vaccines have demonstrated greater induction of germinal center and T cell responses, leading to higher levels of antigen-specific memory B cells, long-lived plasma cells, and antibody titers than recombinant protein vaccines, including in the context of LD vaccination^46,47,75,76^.

crOspC vaccines composed of immunodominant epitopes from diverse OspC types can elicit antibodies that recognize more than 20 distinct OspC types and exhibit borreliacidal activity in vitro^49–52^. Here, we focused on an mRNA vaccine platform encoding OspC along with in-depth mechanistic investigations. We first developed a monovalent OspC mRNA vaccine encoding only OspC type A (OspC-A mRNA) to determine the ability of an mRNA vaccine to protect against homologous infection. The candidate vaccine afforded complete protection against infection and spirochete dissemination to the ear, bladder, heart, ankle joint, and lymph nodes. The vaccine also prevented the development of Lyme carditis and lymph node hyperplasia, as shown through in situ visualization, enumeration of white blood cells, and histopathology^53^.

The OspC-A mRNA vaccine induced significant serum antibody titers with a Th1 bias, indicated by high levels of IgG2a antibodies compared to IgG1 antibodies. The optimal balance between Th1- and Th2-biased responses for *B. burgdorferi* clearance is not clearly established; however, IgG2a antibodies act as opsonins to facilitate ADCP of extracellular bacteria, such as *B. burgdorferi*^77,78^. Accordingly, the antibodies elicited by OspC-A mRNA vaccination induced ADCP in vitro, demonstrating their functionality. Vaccination with an mRNA vaccine targeting OspA previously demonstrated robust IgG titers that persisted for up to 6 months^46^. In this study, we did not investigate the kinetics of the antibody response. As protection against LD has been correlated with antibody titers, further studies will need to be performed to assess the longevity of the protection conferred by OspC mRNA vaccines, especially in comparison to well-established OspA-based vaccines^79,80^.

Next, we examined the antigen-specific cellular immune response to vaccination. In the draining lymph nodes, OspC-A mRNA vaccination induced broad antigen-specific Th1-skewed cytokine responses with high levels of IFN-γ production. CD4 + T cells in the lymph nodes of vaccinated mice also displayed high levels of activation-induced markers (OX40 and CD25) and IFN-γ and IL-2 production in response to stimulation with OspC peptides. Following vaccination, CD4 + T cells may contribute to the clearance of *B. burgdorferi* infection through the induction of *B. burgdorferi*-specific class-switched antibody responses, long-lived plasma cells, and memory B cells^46,81^.

One limitation of OspC-targeting vaccines that must be addressed is the genetic variability of the OspC antigen. Therefore, we developed a prototype polyvalent OspC vaccine containing five of the most common major OspC types (A, C, I, K, and N) in North America. OspC types A, I, K, and N are among the most prevalent OspC alleles detected in human LD infection^54^. In North America, OspC types A, K, C, and N are among the most common OspC types detected in populations of *I. scapularis* ticks^32,55^. We demonstrated that two 1 µg doses of OspC-ACIKN mRNA vaccine elicited significant antibody titers against all five included OspC types and afforded 80-100% protection against infection with *B. burgdorferi* strains expressing OspC types A, I, and K. At the 1 µg dose, significant protection was not observed for type C and N strains, which we have previously shown to be highly infectious^66^. It should be noted that throughout this study, our candidate vaccines displayed an “all-or-nothing” response, in which mice that were protected against infection did not develop carditis and lymphadenopathy, but those that were infected had similar levels of pathology to the control mice. This may be due to the transient nature of OspC expression during infection, potentially suggesting a limited window of vaccine efficacy. In a proof-of-concept follow-up study, we demonstrated that a 3 µg dose of the polyvalent vaccine increased protection against the type C strain from 10 to 50%. Together, these findings suggest that multi-strain protection can be afforded by polyvalent OspC vaccines and that protection against highly infectious strains, such as JD1 (type C) and Bb16-126 (type N) may require dose optimization to achieve full protection. Nevertheless, since ~60% of LD infections are caused by OspC type A or K, the protection demonstrated by the OspC-ACIKN vaccine in this study has the potential to prevent a substantial portion of human LD infections.

One limitation of this study is that the vaccinated mice were challenged by needle injection of spirochetes rather than via tick bite, which is the natural mode of infection. However, previous studies have demonstrated that vaccination with recombinant OspC proteins protects mice against infection via both needles and ticks^29,33,35,48,82,83^. Thus, we expect that our mRNA-based OspC vaccines will protect mice and humans against the bites of *B. burgdorferi*-infected ticks. Anti-OspC antibodies are thought to neutralize the spirochetes in the host at the tick bite site after they had been transmitted by a feeding tick^34^. However, a recent study demonstrated that the infectivity of tick-borne spirochetes (as measured via needle inoculation into naïve mice) was greatly reduced if these ticks had fed on mice that had previously developed a strong antibody response against the homologous strain^34^. This study suggests that anti-OspC IgG antibodies might play an additional role in preventing tick-to-host transmission of *B. burgdorferi* that cannot be captured by needle inoculation studies. Thus, future studies should investigate whether mRNA vaccines protect mice against an infectious tick bite challenge.

In conclusion, our study is the first to demonstrate the efficacy of OspC-targeting mRNA vaccines for LD. Compared to historical LD vaccine designs, our candidate vaccines offer the advantages of targeting OspC with the benefits of the mRNA platform, including an excellent safety profile and robust immunogenicity. To our knowledge, it is also the first study to conduct a systematic challenge of multiple *B. burgdorferi* strains expressing different OspC types for the assessment of a polyvalent OspC vaccine. Future studies should evaluate the efficacy of polyvalent mRNA OspC vaccines in short- and long-term tick challenge models. Ultimately, these results support the advancement of OspC-targeting mRNA vaccines toward clinical development to combat the rising prevalence and health care burden of Lyme disease.

## Methods

### Bacterial cell culture

The *B. burgdorferi* strains used in this paper were characterized in detail in a previous publication, including B31-A3 (OspC type A), JD1 Clone SK143 (OspC type C), Bb16-54 (OspC type I), 297 Ah130 (OspC type K), and Bb16-126 (OspC type N)^66^. Strain B31-A3, and strain B31-A3 engineered to constitutively express OspC (B31-A3-OspC), were kindly provided by Dr. Patricia Rosa (National Institute of Allergy and Infectious Disease, MD, USA)^65,84^. Strain JD1 and strain 297 Ah130 were kindly provided by Dr. George Chaconas (University of Calgary, AB, Canada). Strain Bb16-54 and strain Bb16-126 were obtained from the *B. burgdorferi* isolate collection of the Public Health Agency of Canada. Maintenance of the *B. burgdorferi* strains was performed as previously described^66,85,86^. In brief, *B burgdorferi* was cultured at 35 °C and 1.5% CO_2_ in BSK-H media containing 6% rabbit serum and supplemented with 5 µg/mL Amphotericin B, 100 µg/mL Phosphomycin, and 50 µg/mL Rifampicin. Cultures were grown to mid-log phase and were not subcultured to prevent plasmid loss. Cell counts were determined using a Petroff-Hausser counting chamber under dark-field microscopy.

### Animal care

Female C3H/HeN mice were purchased from Charles River (Senneville, Quebec, Canada). Mice arrived at 6 weeks old and were allowed to acclimatize for 1 week before experimental procedures began. All animal procedures in this study were reviewed and approved by the Health Canada Animal Care Committee and were conducted under protocols #2020-011, #2023-003, and #2024-003 according to institutional guidelines.

### mRNA and LNP synthesis

mRNA encoding firefly luciferase, ospC type A of *B. burgdorferi* strain B31 (GenBank accession #AAC66329.1), ospC type C of strain JD1 (GenBank accession #ADQ31294.1), ospC type I of strain Bb16-54 (GenBank accession #PHOQ01000005.1), OspC type K of strain 297 (GenBank accession #NC_018983.1), or ospC type N of strain Bb16-126 (GenBank accession #PHQD00000000.1) were commercially synthesized by TriLink Biotechnologies (San Diego, CA, USA) with a CleanCap®AG Cap 1 structure and a 120A polyadenylated tail. mRNA was DNAse and phosphatase-treated and purified by a silica membrane. OspC mRNA contained a human tyrosinase signal peptide (MLLAVLYCLLWSFQTSAGHFPRA; GenBank accession #AH003020) at the N-terminus. All mRNA was fully substituted with N1-methyl-pseudo-uridine and was codon optimized for expression in mice.

LNPs were produced using a NanoAssemblr BT™ or Ignite™ instrument (Precision Nanosystems, Inc., Vancouver, BC) as described previously, encapsulating mRNA instead of plasmid DNA^85^. The percent molar ratio was 50:10:38.5:1.5, respectively, of lipids heptadecan-9-yl-8-((2-hydroxyethyl)(6-oxo-6-(undecyloxy)hexyl)amino)octanoate (SM-102) (MedKoo Biosciences, Morrisville, NC, USA), 1,2-distearoyl-*sn*-glycero-3-phosphocholine (DSPC, Avanti Polar Lipids, Inc., Alabaster, AL, USA), ovine cholesterol (Millipore Sigma, Burlington, ON, CA), and 1,2-dimyristoyl-rac-glycero-3-methoxypolyethylene glycol-2000 (DMG-PEG2000, Millipore Sigma). Lipids in ethanol were mixed at a ratio of 3:1 with aqueous 50 mM sodium citrate, pH 4, containing the mRNA at an N/P ratio of 6. For empty LNP controls, the aqueous phase contained 50 mM sodium citrate only.

### LNP characterization

LNP size was characterized by nanoparticle tracking analysis (NTA) using a NanoSight 300 instrument (Malvern Panalytical, Westborough, MA, USA) as described previously^85^. Average particle size ranged from 68 to 83 nm. The efficiency of nucleic acid encapsulation was determined using Triton X-100 (Millipore Sigma) and SYBR™ Gold dye (Thermo Fisher, Ottawa, ON, CA) as described previously^85^. In brief, LNPs were disrupted with 1% Triton X-100 in a 96-well plate, and nucleic acids were quantified by the addition of 1X SYBR™ Gold dye and measured using a Synergy MX plate reader (Ex/Em: 495/537 nm) (BioTek, Winooski, Vermont, USA). Disrupted LNPs were measured alongside undisrupted LNPs and a nucleic acid standard curve. The encapsulation efficiency was determined by subtracting the unencapsulated mRNA from the total amount of mRNA in a sample and then dividing by the total amount of mRNA. The encapsulation efficiencies ranged from 88 to 98%.

### Mass spectrometry confirmation of OspC expression

Expression of the OspC proteins was confirmed by mass spectrometry following transfection of HEK293T cells in vitro with OspC-ACIKN mRNA encapsulated in LNPs (Fig. S3). HEK293T cells were incubated with 500 ng of OspC-ACIKN mRNA encapsulated in LNPs at 37 °C and 5% CO_2._ After 24 h, the cells were lysed in Radio-Immunoprecipitation Assay buffer and then pulse sonicated. The samples were processed with a single pot solid phase-enhanced sample preparation (SP3) strategy, consisting of reduction with 10 mM DTT followed by alkylation with 20 mM IAA. Protein was bound to SeraMag Magnetic carboxylate beads in the presence of 80% ACN, then impurities were removed with three washes of 80% ACN. Trypsin was added at a ratio of 1:25 enzyme: protein and incubated overnight at 37 °C.

Tryptic peptides were analyzed using an Orbitrap Astral Mass Spectrometer coupled with a Vanquish Neo UHPLC System (Thermo Fisher Scientific Inc.). For each injection, 1 ul (500 ng peptides) were analyzed by loading onto a PepMap Neo C18 trap and desalting with 0.1% formic acid in water (solvent A) before separating on a 75 μm I.D. × 150 mm fused silica analytical column packed in-house with 3-μm ReproSil-Pur C18 beads (100 Å; Dr. Maisch GmbH, Ammerbuch, Germany). Chromatographic separation was achieved at a flow rate of 500 nl/min. The gradient was set as 5–45% buffer B in 28.5 min, the spray voltage was set to 2.6 kV, and the temperature of the heated capillary was 300 °C. Scan range was from 380 to 980 m/z for both MS and DIA. The DIA window was set to 2 Da, with HCD energy of 25%. The mass resolution was 240,000 for ms1. A real-time internal calibration by the lock mass was used. All data were recorded with Xcalibur software (Thermo Fisher Scientific, San Jose, CA).

The software package DiaNN (v2.1.0) was used to analyze the data with a library-free search against the reviewed Uniprot Human database (downloaded 20,241,120, entries) containing the sequences of the five OspC types added. The resulting protein and peptide results were filtered to view only OspC. Relative abundance was manually calculated by the Top3 method by averaging the three most abundant precursors, ignoring shared peptides. The relationship between MS signal response and protein concentration is constant within a CV of less than ±10%.

### Immunizations and bacterial challenge

For the monovalent protection study, 8-week-old female C3H/HeN mice were randomly assigned to groups and injected intramuscularly in the hind leg with LNPs containing 1 µg of mRNA encoding OspC-A (OspC-A mRNA, *n* = 5) or an empty LNP negative control containing a matched amount of lipid (Control, *n* = 5) (Fig. 1A). For the polyvalent protection study, 8-week-old female C3H/HeN mice were randomly assigned to groups and injected intramuscularly with LNPs containing 3 µg of each mRNA encoding the five OspC types (OspC-ACIKN, *n* = 10) or 3 µg of mRNA encoding firefly luciferase (Control, *n* = 10) (Fig. 6A). The total injection volume was 50 µL divided between the two hind legs. Mice received an identical boost injection 4 weeks later and were bled 3 weeks after the boost injection for the collection of serum. Bacterial challenge was performed 4 weeks after the boost injection by subcutaneous needle injection between the shoulder blades.

For the challenge, spirochetes were grown to mid-log phase, centrifuged at 5000×*g* for 20 min, and resuspended in 100 µL of incomplete BSK-H media without rabbit serum. For the monovalent challenge, mice were injected with 10^6^ spirochetes of strain B31-A3. For the OspC-ACIKN vaccine study challenge, mice were injected with 10^6^ spirochetes of strain B31-A3 (OspC type A), Bb16-54 (OspC type I), or 297 Ah130 (OspC type K), or 10^4^ spirochetes of strain JD1 (OspC type C) or Bb16-126 (OspC type N). Challenge doses were determined based on our previous study characterizing strain infectivity^66^. An additional group of five mice per study were injected with phosphate-buffered saline (PBS) instead of LNPs at day 0 and 28 and incomplete BSK-H media without bacteria instead of challenge at day 56 to serve as naive controls for histopathological analysis. Fourteen days after the challenge (d70), mice were euthanized by cardiac puncture exsanguination and cervical dislocation under isoflurane anesthesia for sample collection.

For the immunogenicity study, 8-week-old female C3H/HeN mice were randomly assigned to groups and injected intramuscularly with either 1 µg of mRNA encoding OspC-A encapsulated in an LNP (OspC-A mRNA, *n* = 10) or 1 µg of mRNA encoding firefly luciferase encapsulated in a LNP (Control, *n* = 10). Mice received an identical boost injection 4 weeks later and were euthanized 4 weeks after the boost for sample collection. Euthanasia was performed by cardiac puncture exsanguination and cervical dislocation under isoflurane anesthesia.

### Tissue culture for bacterial identification

Live spirochetes can be cultured from mouse tissues to determine the mouse infection status and spirochete viability. The skin surrounding the bacterial injection site and the right ear were collected 2 weeks after challenge and cultured in complete BSK-H media for 28 days at 35 °C and 1.5% CO_2_. Cultures were examined under dark-field microscopy across five fields of view and were considered positive if at least one motile spirochete was observed.

### Quantitative PCR (qPCR) for bacterial burden

Two weeks after the bacterial challenge, the mice were euthanized, and the bladder, left ear, left tibiotarsal joint, inferior half of the heart, and left inguinal and medial iliac lymph nodes were collected and frozen in liquid nitrogen. DNA extraction from mouse tissues was performed using a DNAeasy kit (Qiagen, Toronto, ON, CA) according to the manufacturer’s instructions and qPCR analysis was performed as described previously, targeting the *flaB* gene of *B. burgdorferi* and standardized per 10^6^ mouse β-actin gene copies^85^.

### Determination of lymphadenopathy

Quantification of lymphadenopathy was performed as described previously^86^. Briefly, the left axillary lymph nodes were collected 2 weeks after bacterial challenge, pressed between two microscope slides, and filtered through a 70-µm cell strainer to create a single-cell suspension. White blood cell counts were determined using a Sysmex XT-2000iV hematology analyzer.

### Histopathology

Histopathology analysis of the heart and axillary lymph nodes was performed as described previously^85,86^. In brief, the superior half of the heart and the right axillary lymph nodes were collected 2 weeks after bacterial challenge into 10% neutral buffered formalin for routine histopathological processing and hematoxylin and eosin staining. Inflammation in the heart tissue and lymph nodes was graded on a scale from 0 to 5 based on International Harmonization of Nomenclature and Diagnostic Criteria for Lesions in Rats and Mice (INHAND) standards, where 0 was normal, 1 was minimal, 2 was mild, 3 was moderate, 4 was marked, and 5 was severe^87,88^. Lymph nodes were graded individually 0–5 for follicular hyperplasia, paracortical hyperplasia, medullary cord plasma cell hyperplasia, and the combined pathology score represents the sum of these three grades.

### Enzyme-linked immunosorbent assay (ELISA)

OspC-specific antibody titers were quantified according to the method described previously^85,86^. Briefly, recombinant OspC type A, C, I, K, or N were used to coat 96-well Nunc Maxisorp™ flat-bottom plates (Thermo Fisher) at a concentration of 1 µg/mL in 100 µL overnight at 4 °C. Plates were blocked with 1% (w/v) bovine serum albumin (IgG-Free, Protease-Free, Jackson ImmunoResearch, West Grove, PA) and incubated with serum collected 4 weeks after boost vaccination, serially diluted in PBS containing 0.05% Tween-20 (PBS-T) for 1 h at 37 °C. For total IgG ELISAs, the serum dilutions were twofold from 1:50 to 1:102,400. For IgG1 and IgG2a ELISAs, the serum dilutions were three-fold from 1:50 to 1:8,857,350. Following serum incubations and washing, HRP-conjugated goat anti-mouse IgG (Cytiva, Marlborough, MA), HRP-conjugated goat anti-mouse IgG1 (Jackson ImmunoResearch), or HRP-conjugated goat anti-mouse IgG2a (Jackson ImmunoResearch) was added to each plate at a 1:5000 dilution in PBS-T. Tetramethyl-benzidine (TMB) substrate (Cell Signaling Technology, Danvers, MA, USA) was added to each well, followed by 0.16 M sulfuric acid, and then the absorbance was read at 450 nm using a spectrophotometer. Endpoint titers were defined as the reciprocal of the highest dilution that resulted in an optical density (OD) greater than the average OD of all wells containing serum from control mice plus three times the standard deviation.

### Antibody-dependent phagocytosis (ADCP) assay

ADCP activity was measured as previously described^86^. In brief, mouse serum samples were collected 4 weeks after boost vaccination, diluted 1:10 in Dulbecco’s Modified Eagle Medium (DMEM) containing no serum or antibiotics, and heat-inactivated at 56 °C for 30 min. In a 96-well plate, 20 µL of diluted inactivated serum was incubated with *B. burgdorferi-*OspC stained with a FITC-conjugated anti-Borrelia polyclonal antibody (PA1-73005, Thermo Fisher) at a concentration of 3.75 × 10^7^ cells/mL for 15 min at 37 °C and 300 rpm to enable opsonization. Then, 10 µL of RAW 264.7 cells (ATCC TIB-71), a mouse macrophage cell line, at a concentration of 7.5 × 10^6^ cells/mL were added to each well before shaking for 15 min at 37 °C and 300 rpm and then incubating for 1 h at 37 °C without shaking. Phagocytosis was arrested by the addition of 100 µL of cold PBS, and ADCP activity was represented by the percentage of FITC + RAW 264.7 cells using a BD FACSymphony™ A1 flow cytometer.

### Serum borreliacidal assay

Serum borreliacidal activity was measured as previously described^85^. In brief, 4 µL of mouse serum samples collected 4 weeks after boost vaccination were incubated with 4 µL of mid-log phase *B. burgdorferi-*OspC culture diluted to 5 × 10^7^ cells/mL and 4 µL of guinea pig serum (GPS, Complement Technologies, Tyler, Texas, USA) for 24 hours at 35 °C and 1.5% CO_2_. Motile cells were then quantified using a Petroff-Hausser counting chamber under dark-field microscopy, and the percentage of live spirochetes was determined by dividing the cell count by the average cell count of control samples without serum.

### Generation of single-cell suspensions from the draining lymph nodes

Four weeks after OspC-A mRNA or Control boost vaccination, the draining popliteal and inguinal lymph nodes were collected in RPMI media. The nodes were gently squished between two microscope slides and passed through a 70 µm cell strainer before centrifugation at 400×*g* for 5 min. Cells were resuspended in RPMI, and white blood cells were counted using a Sysmex XT-2000iV hematology analyzer.

### Multiplex cytokine ELISA

The multiplex cytokine ELISA was performed using a ProcartaPlex 17-plex Immunoassay kit (EPX170-26087-901, Thermo Fisher) as described previously^86^. In brief, 1 million lymph node cells in single-cell suspension from OspC-A vaccinated or control mice (*n* = 5 per group) were stimulated with a custom overlapping peptide pool of OspC type A (GenScript, Piscataway, NJ, USA) for 24 h at 37 °C before centrifugation at 400×*g* for 5 min. The supernatant was collected and frozen at −80 °C until the assay was performed according to the manufacturer’s instructions. A Luminex 200 system (Millipore Sigma) was used to read the plate, and MILLIPLEX Analyst software version 5.1 was used to determine the concentration of each cytokine.

### Flow cytometry of activation-induced markers

In a 96-well plate, 1 million mouse lymph node cells (*n* = 5 per group) in single-cell suspension from OspC-A vaccinated or control mice were stimulated individually with a custom 15-mer overlapping peptide pool of OspC type A containing 1 µg of each peptide (GenScript) in RPMI media for 18 h at 37 °C, 5% CO_2_. Unstimulated lymph node cells were included as a negative control, and lymph node cells stimulated with phorbol 12-myristate-13-acetate (Sigma-Aldrich, St. Louis, MO, USA) at 20 ng/mL and ionomycin (Sigma-Aldrich) at 1 µg/mL (PMA+ionomycin) were included as a positive control. Following the incubation, lymph node cells were centrifuged at 500×*g* for 5 min, washed twice with FACS buffer, and resuspended in FACS buffer containing rat anti-mouse CD16/CD32 (Mouse Fc Block™, BD Biosciences, Mississauga, ON, CA). The plate was incubated for 15 min at 4 °C and then centrifuged at 500×*g* for 5 min. The lymph node cells were resuspended in antibody cocktail containing anti-CD3 Alexa Fluor® 700 (Clone 17A2, BD Biosciences), anti-CD4 BB700 (Clone RM4-5, BD Biosciences), anti-OX40 BV786 (Clone OX-86, BD Biosciences), anti-CD25 PE (Clone OX-39, BD Biosciences), and LIVE/DEAD™ Fixable Blue Dead Cell Stain (ThermoFisher) diluted in Brilliant Stain Buffer (BD Biosciences) and incubated for 30 min at 4 °C in the dark. Lymph node cells were washed twice with FACS buffer, fixed in Cytofix™ fixation buffer (BD Biosciences) for 15 min at room temperature, washed twice again and then resuspended in FACS buffer. Acquisition was performed using a BD FACSAria™ Fusion flow cytometer, and data analysis was performed using FlowJo v10 software (Treestar) according to the gating strategy depicted in Fig. S4.

### Enzyme-linked immunosorbent spot (ELISpot) assay

The ELISpot assay was performed using a Mouse IFN-γ/IL-4 Double-Color ELISPOT kit according to the manufacturer’s instructions (ImmunoSpot by Cellular Technology Limited, Shaker Heights, Ohio, USA). For stimulation, 1 million lymph node cells in single-cell suspension from OspC-A vaccinated or control mice (*n* = 5 per group) were stimulated individually with a custom 15-mer overlapping peptide pool of OspC type A containing 1 µg of each peptide (GenScript). The plates were read using an ImmunoSpot® S6 Analyzer, and data analysis was performed using ImmunoSpot® v7.0 software.

### Intracellular cytokine staining

Intracellular cytokine staining and flow cytometry analysis of antigen-specific T cells was performed as described previously using mouse lymph node cells instead of splenocytes^86^. In brief, 1 million lymph node cells in single-cell suspension from OspC-A vaccinated or control mice (*n* = 5 per group) were stimulated individually with a custom 15-mer overlapping peptide pool of OspC type A containing 1 µg of each peptide (GenScript) for 5 h. Unstimulated and PMA+ionomycin-stimulated samples were included as negative and positive controls, respectively. GolgiPlug™ (Brefeldin A, BD Biosciences) was added to each well after the first hour. After the 5 h incubation, lymph node cells were washed with PBS, blocked with rat anti-mouse CD16/CD32 (Mouse Fc Block™, BD Biosciences), and surface stained for 30 min at 4 °C with anti-CD3 Alexa Fluor® 700 (Clone 17A2, BD Biosciences), anti-CD4 BB700 (Clone RM4-5, BD Biosciences), anti-CD8a FITC (Clone 53-6.7, BD Biosciences) and LIVE/DEAD™ Fixable Blue Dead Cell Stain (Thermo Fisher) diluted in Brilliant Stain Buffer (BD Biosciences). Lymph node cells were fixed and permeabilized before intracellular staining with anti-IFN-γ BV786 (Clone XMG1.2, BD Biosciences), anti-TNF BV650 (Clone MP6-XT22, BD Biosciences), and anti-IL-2 (Clone JES6-5H4, BD Biosciences) diluted in Brilliant Stain Buffer (BD Biosciences) for 30 min at 4 °C. Cells were resuspended in PBS and acquired immediately using a BD FACSAria™ Fusion flow cytometer. Data analysis was performed using FlowJo v10 software (Treestar) according to the gating strategy described previously^86^.

### Statistical analysis

Statistical significance of data or log10-transformed data were calculated using unpaired two-tailed *t*-tests or Fisher’s exact test at a significance value of α = 0.05. All statistical analyses were performed using GraphPad Prism 9 (Dotmatics Inc., Boston, MA, USA). ns not significant, *p value <0.05, **p value <0.01, ***p value <0.001, ****p value <0.0001.

## Supplementary information

41541_2025_1326_MOESM1_ESM.pdf

## Data Availability

The raw data supporting the conclusions of this article will be made available by the authors on request.
