# Supplementary material for: Polyvalent mRNA vaccine targeting outer surface protein C affords multi-strain protection against Lyme disease
Source: NPJ Vaccines. 2025 Dec 4;11:4. doi: 10.1038/s41541-025-01326-3 (PMC12774967; doi:10.1038/s41541-025-01326-3)
Supplement: Supplementary file 1 — 41541_2025_1326_MOESM1_ESM.pdf [file 41541_2025_1326_MOESM1_ESM.pdf]

Supplemental Materials

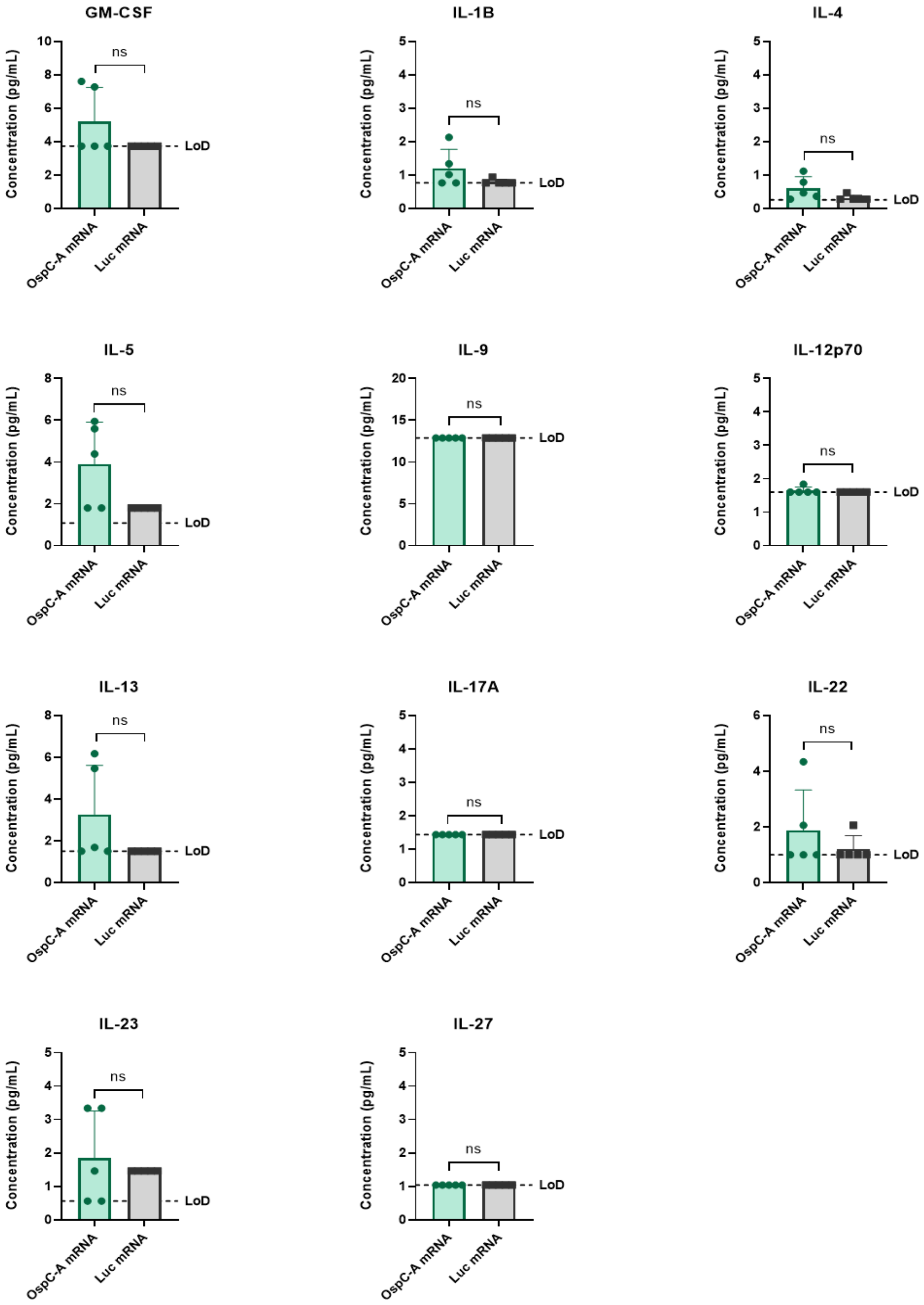

Figure S1. Cytokines that were not significantly elevated in the OspC-A mRNA group following stimulation of the draining lymph nodes with an OspC overlapping peptide pool. dLNs were

harvested from mice four weeks after boost vaccination with the OspC-A mRNA vaccine or Control. dLNs were stimulated with an overlapping peptide library of OspC for 24 h. The concentration of each cytokine in the supernatant was determined by a ProcartaPlex 17-plex Immunoassay kit. LoD, limit of detection. Error bars represent standard deviation. Statistical significance was calculated using unpaired two-tailed t-tests. ns, not significant.

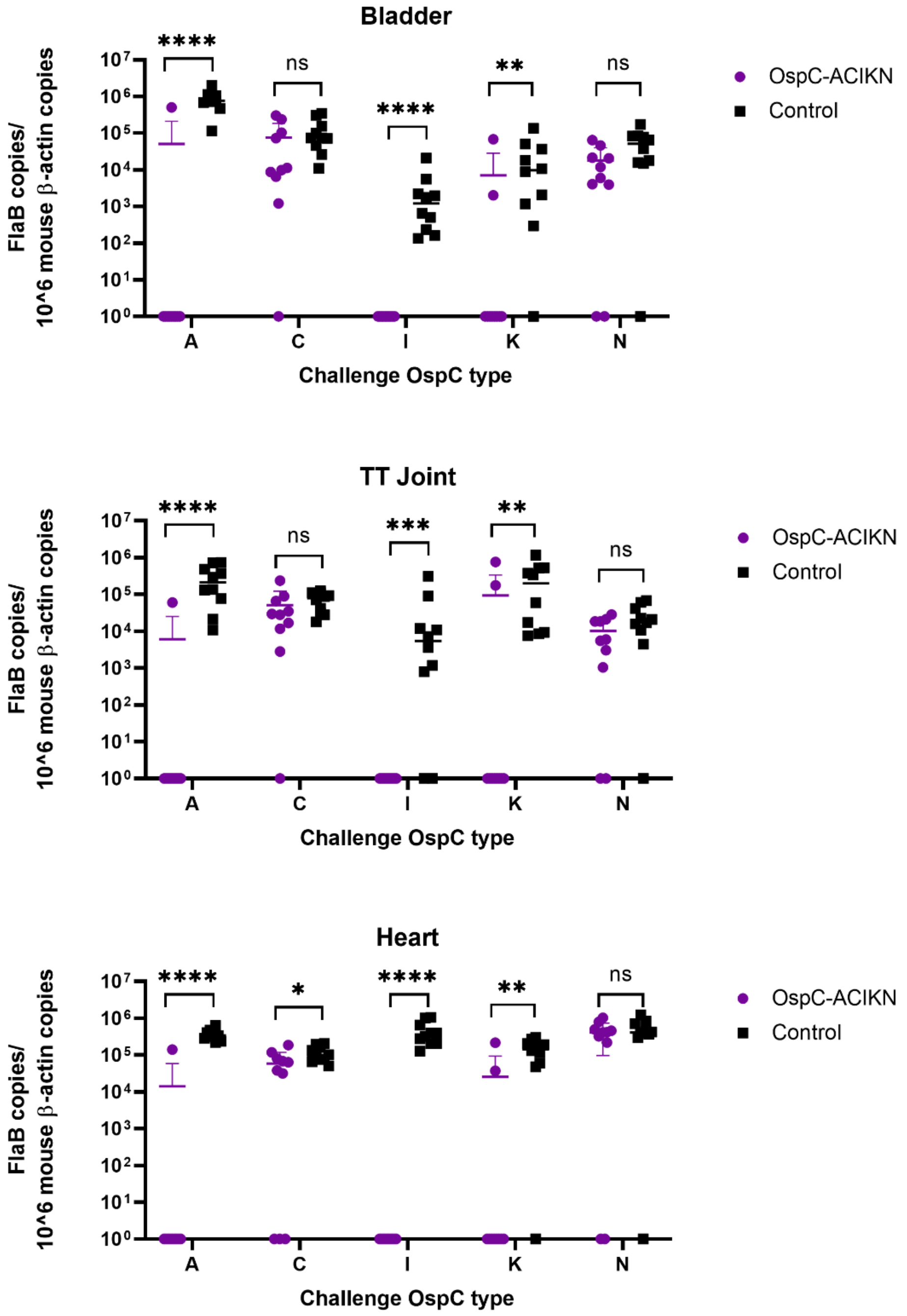

Figure S2. Vaccination with OspC-ACIKN mRNA protects mice against disseminated infection with multiple *B. burgdorferi* strains. qPCR analysis of the bacterial DNA burden in the bladder

(top), tibiotarsal joint (TT joint) (middle) and heart (bottom) represented by *B. burgdorferi* FlaB gene copies per 10^6 mouse β-actin copies. Samples below the threshold of amplification are represented by a value of 1. Statistical significance was calculated using independent two-tailed t-tests. Error bars represent standard deviation. * p-value < 0.05, ** p-value < 0.01, *** p-value < 0.001, **** p-value < 0.0001.

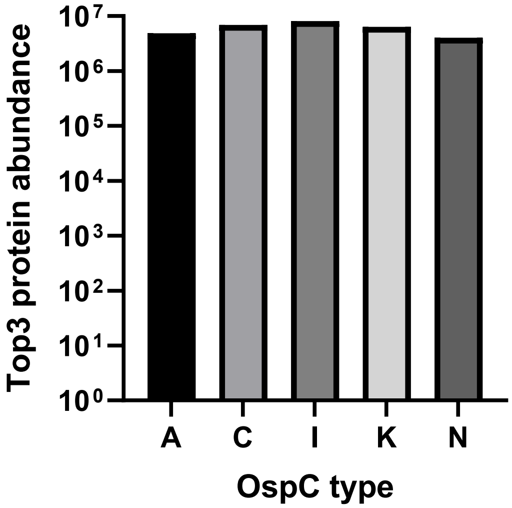

Figure S3. Protein abundance by OspC type following *in vitro* transfection of HEK 293T cells with LNPs encapsulating OspC-ACIKN.

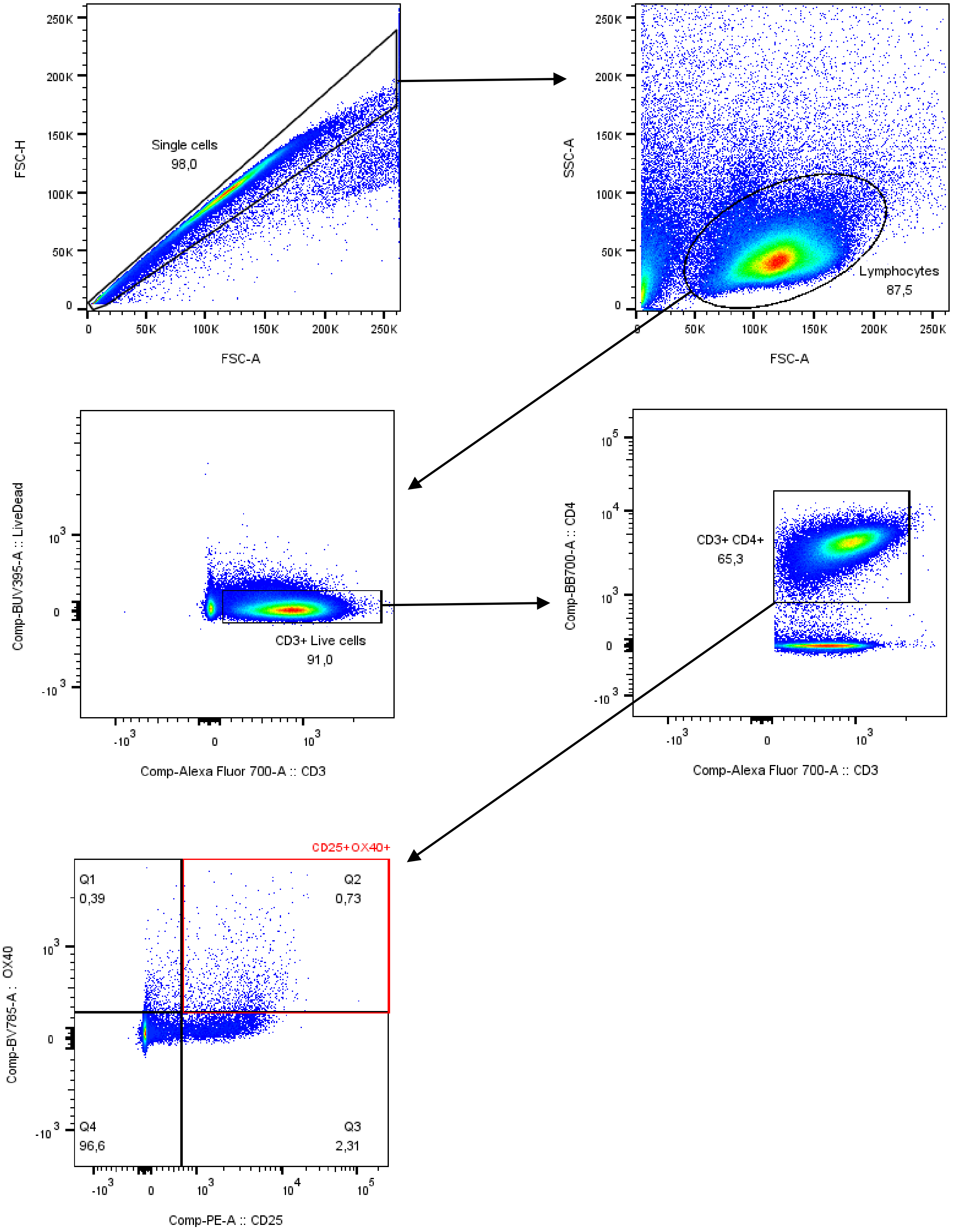

Figure S4. Representative gating strategy for the activation induced marker (AIM) flow cytometry assay. Activated T cells were CD4+ CD25+ OX40+.
